# Childhood intussusception in Uzbekistan: Analysis of retrospective surveillance data

**DOI:** 10.1186/1471-2431-11-22

**Published:** 2011-03-24

**Authors:** Renat Latipov, Rajabboy Khudoyorov, Elmira Flem

**Affiliations:** 1 Reference Laboratory, Tashkent, Uzbekistan; 2 Scientific Center for Emergency Medicine, Bukhara, Uzbekistan; 3 Division of Infectious Disease Control, Norwegian Institute of Public Health, Oslo, Norway

## Abstract

**Background:**

Estimates of baseline incidence of childhood intussusception could help safety monitoring after the introduction of rotavirus vaccines. We studied the incidence of intussusception in Uzbekistan, a GAVI-fund eligible state in Central Asia.

**Methods:**

We retrospectively reviewed intussusception cases in children <2 years of age treated during 2004-2008 at 15 hospitals in the Bukhara region of Uzbekistan. Demographic and clinical data as well as information on diagnostic and treatment practices were obtained from hospital records. We categorized cases using the Brighton collaboration clinical case definition and calculated the national incidence rate.

**Results:**

Over a 5-year study period, 67 confirmed cases were identified, of which 67% were boys. The median age was 12 months, and no seasonal trend in the distribution of cases was observed. The diagnostic methods used included abdominal radiography (87%) and ultrasonography (57%). Intussusception reduction by air enema was successful in 33 (49%) patients and 34 (50%) cases underwent surgery. A total of 4 deaths occurred, including 3 deaths in infants aged 0-6 months. The median length of hospital stay was 7.3 (range 0-37) days. The incidence of intussusception is estimated at 23 (95% CI 13.6-32.4) cases per 100,000 child-years, corresponding to approximately 237 cases annually.

**Conclusions:**

This is the first study to estimate the incidence of childhood intussusception prior to the introduction of the rotavirus vaccination in Uzbekistan. A prospective surveillance system using a standardized case definition is needed in order to better examine the occurrence of intussusception in developing countries.

## Background

Intussusception (IS) is the most common cause of bowel obstruction in young children, which involves an invagination of a proximal segment of the intestine into a distal segment. Intussusception is characterized by a sudden onset of abdominal pain, vomiting, rectal bleeding, and the presence of a palpable abdominal mass [1]. The condition is diagnosed by ultrasonography, radiology or surgery, and is usually treated by using air or hydrostatic reduction enema under radiologic or ultrasound guidance. However, surgery may be required in some cases, and approximately 10% of patients with IS undergo an intestinal resection due to a vascular injury to the intestine [2]. Intussusception primarily affects children, with the peak incidence reported at between 4 to 10 months of age [2], although adults cases are reported as well [1]. The background incidence of IS varies from 0-17.8 cases per 100,000 children [3-5] to 302 cases per 100,000 children [6,7] across various regions, with a recently decreasing trend in IS incidence reported in the industrialized countries [8]. Case-fatality rates also vary widely by region, and deaths from IS are more common in developing settings than in industrialized countries [9].

Although the full etiology of IS remains unclear [1,10], adenovirus infection has been reportedly associated with an increased risk of IS [11]. However, natural rotavirus infection is not believed to be linked to this condition[11], but an association of IS with an oral rhesus-based tetravalent rotavirus vaccine is well documented [12]. This vaccine was licensed in the USA in 1998 and administered to approximately 500,000 infants; the subsequent risk of IS was estimated to be one case per 10,000 vaccine recipients [13]. In view of this association, the vaccine was withdrawn from the market by the manufacturer. In 2004, a new rotavirus vaccine (Rotarix^®^, GSK) was licensed in Mexico [14] and in 2006, another vaccine (Rotateq^®^, Merck) was approved in US [15]. At present, both vaccines are used in routine immunization in several high and low-income countries. Clinical trials of these vaccines and early post-licensure data [16,17] demonstrated no major risk of IS after vaccination (<1 case in 20,000 vaccines). Even so, the risk of a lower magnitude could not be entirely ruled out before further post-licensure data are accumulated. For that reason, local data on the baseline incidence and epidemiology of IS are important for countries that are considering using rotavirus vaccines to help with post-introduction safety monitoring.

As in many other developing countries, such data are not available in Uzbekistan, a GAVI-fund eligible country in Central Asia with 650,000 annual births [18]. It is estimated that at least 30% of all hospitalizations for acute gastroenteritis in Uzbek children <5 years of age are attributable to rotavirus, and 1,174-1,857 rotavirus deaths in children <5 years old occur annually [19]. In case of rotavirus vaccine introduction in Uzbekistan, monitoring IS as a part of the surveillance of vaccine-associated adverse events would be challenging since no IS surveillance is currently conducted. In addition, a limited amount of previous reports do not allow for the establishment of reliable baseline rates for IS among children [20]. Therefore, our aim is to estimate the incidence and describe the epidemiology of IS in young children before vaccine introduction in order to assist future safety monitoring. This study is based on methods described in the World Health Organization (WHO) guidelines for the post-marketing surveillance of rotavirus vaccine safety [21] and applies the internationally standardized criteria for defining IS as per the recommendations of the Brighton Collaborations Working Group [22].

## Methods

### Study area and participating hospitals

Uzbekistan is administratively divided into 12 regions, and we selected the Bukhara region in the central part of the country as the surveillance area for this study. This region was chosen based on WHO guidelines which recommended a surveillance area with a population of at least 50,000 infants to establish IS incidence with a 95% confidence interval of 1.0-3.7 per 10,000 population [23]. The region includes 11 districts and the 2 cities of Bukhara and Kagan; the total population of the region was 1,561,338 in 2008 with 59,238 being children <2 years of age (source: Ministry of Health). The Uzbek health care system is state-owned and includes outpatient clinics and hospital facilities at the local and district levels, in addition to tertiary hospitals at the regional level. Patients with IS are usually admitted and managed in the surgical ward of district hospitals with a majority of cases treated in the district they reside in. Sometimes cases may be admitted to hospitals in other districts within the same region or to regional hospitals. In the Bukhara region, a total of 15 hospitals (11 district hospitals, 2 hospitals in Bukhara, 1 hospital in Kagan, and the Regional Center for Emergency Surgery in Bukhara) diagnose and treat IS cases. In order to maximize the number of case findings, all 15 hospitals were included in this study. Permission to use data in the study was received from the participating hospitals.

### Case definition and data sources

Children <24 months of age who were diagnosed with IS at one of the 15 hospitals in the Bukhara region from 1 January 2004 through 31 December 2008 were eligible for the study. Using the case definition developed by the Brighton Collaboration Working Group [24], we classified all identified cases into confirmed, probable and suspected, and used 3 levels of diagnostic certainty. Patients at Level 1 of diagnostic certainty were defined as confirmed cases. Level 1 requires one of the following: demonstration of invagination of the intestine at surgery and/or by either air or liquid-contrast enema, presence of intra-abdominal mass on ultrasonography, and/or the demonstration of invagination at autopsy. Cases diagnosed using a combination of clinical symptoms and signs according to Levels 2 and 3 of diagnostic certainty were defined as probable. Suspected cases were patients with a diagnosis of IS for whom the available information did not allow for a determination of the level of diagnostic certainty. Data for each identified case were collected by reviewing admission and discharge logs, case history records, ultrasonography, radiology logs, and surgery reports from the respective hospitals.

### Data collection and analysis

For each identified child, we extracted information on demographics, admission and discharge dates, clinical signs and symptoms and their duration, as well as diagnostic and treatment procedures performed. Symptoms and signs were recorded as positive or negative only if the presence or absence of the symptom or sign was documented by the medical and/or nursing staff in the patient's records. Data were summarized on the standardized questionnaire, entered into an electronic database and checked for accuracy, and the data extraction and entry were performed by the same investigator. We analyzed the information according to age, sex, clinical signs, year and month of hospitalization, and diagnostic and treatment-related characteristics. Because early diagnosis and treatment of IS could provide better outcomes, we compared treatment outcomes and the length of hospital stay for patients admitted <24 hours and ≥24 hours after the onset of symptoms. The prevalence rate was calculated by using the following formula: result of the ratio of number of intussusception to the number of children under the age of 2 years for each year in Bukhara region, followed by calculation of the average prevalence rate for 5 years. Based on the census data (source: Ministry of Health) and the prevalence of IS in the Bukhara region the estimated number of IS for whole Uzbekistan was calculated. A statistical analysis was conducted by using STATA Version 10 (STATACorp LP, College Station, Texas, USA). Categorical variables were compared by Mantel-Haenzel and Fisher's exact tests, and continuous variables were compared by an analysis of variance or the Kruskal-Wallis test. All statistical tests were two-tailed, and a P-value of <0.05 was considered significant. For this study the approval of Ethics committee of Ministry of Health of Uzbekistan and the Ethics committee for medical research in Norway had received.

## Results

**Figure 1:**
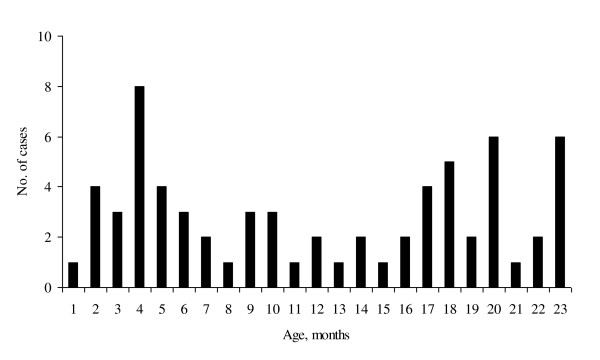
**Age distribution of intussusception in 67 children aged <24 months, Bukhara region, Uzbekistan, 2004-2008**.

During 5 years, 67 confirmed cases of IS in children <24 months of age were identified, corresponding to an incidence of 23 cases [95% CI 13.6-32.4] per 100,000 child-years or approximately 237 cases per year. A slightly higher incidence among children 0-6 months of age that will be targeted by rotavirus vaccination was observed (Table 1). Among confirmed cases, boys (67%) were significantly (p < 0.05) younger than girls (10.4 vs. 15.4 months), and the median age was 12.1 months. We found a biphasic age distribution with the fist peak in children aged 3-6 months and the second peak among those 18 months and older (Figure 1).

**Table 1:** Incidence of intussusception-associated hospitalizations in children aged <24 months, Bukhara region, Uzbekistan, 2004-2008

Age, months	Boys		Girls		Total	
	
	n (%)	**Rate **^**a **^**(95%CI)**	n (%)	**Rate **^**a **^**(95%CI)**	n (%)	**Rate **^**a **^**(95%CI)**
0-6	19 (42.2)	46.1 (32.9-59.5)	4 (18.2)	9.7 (3.7-15.9)	23 (34.3)	27.9 (17.6-38.3)
7-12	9 (20.0)	27.3 (17.1-37.6)	3 (13.6)	9.1 (3.2-15.1)	12 (17.9)	18.2 (9.9-26.6)
13-18	9 (20.0)	25.2 (15.4-35.0)	6 (27.3)	16.8 (8.8-24.9)	15 (22.4)	21.0 (12.0-30.0)
19-24	8 (17.8)	22.4 (13.2-31.7)	9 (40.9)	25.2 (15.4-35)	17 (25.4)	23.8 (14.3-33.4)
Total	45	30.9 (20.0-41.8)	22	15.1 (7.5-22.8)	67	23.0 (13.6-32.4)

^a ^Rates per 100,000 child-years.

**Figure 2:**
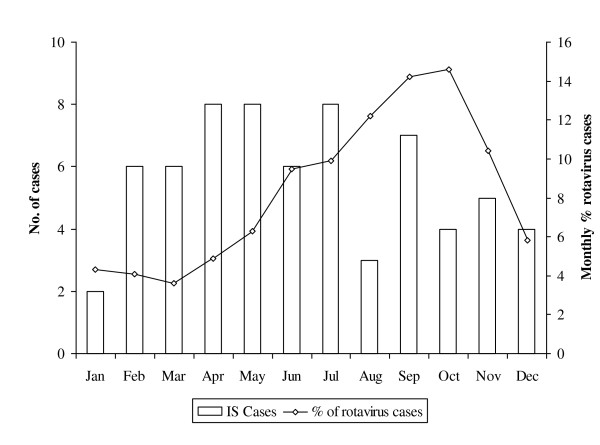
**Monthly distribution of intussusception in children aged <24 months, Bukhara region, Uzbekistan, 2004-2008**.

The most frequent symptoms were abdominal pain (97%), palpable intestinal mass (97%), and bile-stained vomiting (80.6%). The lethargy (83.6%) and pallor (64.2%) were also observed frequently. Detection of blood on rectal examination (82.1%) and presence of IS (79.1%) or fluid level and dilated loops on plain abdominal radiography (20.9%) were the main symptoms for diagnosis. A rectal mass was detected in 3 cases (4.5%), and the presence of "red currant jelly" stool was only reported in a single case. The classic triad of vomiting, passage of blood through the rectum and abdominal pain was documented in 8 (11.9%) of 67 children. The clinical presentation at admission also included symptoms of concurrent gastroenteritis in 36% of children and concurrent respiratory symptoms in 49% of cases. We did not detect any significant differences in the distribution of symptoms by age or sex. No statistically significant changes in the monthly occurrence of IS were found despite a potential increase in the number of cases during April and May (Figure 2). Importantly, no increase was observed during autumn when most rotavirus disease occurs in Uzbekistan [19].

The most frequent diagnostic methods were plain abdominal radiography (86.6%) and ultrasonography (56.7%); diagnosis was made at surgery in 5 cases (7.5%), and in 2 cases, IS was first diagnosed clinically and later confirmed at surgery. The frequency of using various diagnostic procedures did not differ among the study hospitals. A reduction of IS by air enema was attempted in 60 patients, but was successful in only 33 (49.3%) children. Thirty four (50.7%) cases underwent surgery, of which seven patients did not have previous air enema. These patients were admitted >24 hours after symptoms onset and had features of intestinal vascular compromise or venous congestion such as passage of blood per rectum or blood on rectal examination.

A reduction by air enema was used less often in younger children, and surgery rates were more frequent in children aged 0-6 months (60.9%) compared with older children (45.5%). Overall, 4 deaths occurred among the 67 patients which yielded a case-fatality rate of 6% with a higher rate in infants aged 0-6 months compared to older children (15% vs 2.3%). The mean length of hospital stay was 7.3 days, but patients undergoing surgery stayed in the hospital 5 times longer (12.3 days) than patients with a successful reduction by air enema (2.2 days) (p < 0.001). It is known that the earlier a child arrives at the hospital after the onset of illness, the higher the frequency of successful conservative treatment [1]. In our study, air enema reduction was more likely to be successful in patients presented within 12 hours following the onset of symptoms (a 72.7% success rate), with no deaths reported in such cases. By comparison, a reduction by air enema was successful in only 59.6% of patients treated within 24 hours after symptom onset, with a single death occurring in this group. Among children admitted within the first 24 hours after the onset of illness, no intestinal resection was performed. However, 4 of 9 children admitted >24 hours after symptom onset had intestinal resection, with 3 deaths in these patients compared to a single death in children admitted earlier to the hospital.

## Discussion

The withdrawal of the first rotavirus vaccine from the market due to the increased risk of IS in vaccine recipients emphasized the need for a careful assessment of the safety of new vaccines. Although available evidence to date does not indicate the risk of IS after vaccination, a vigilant post-marketing surveillance is recommended to rule out such risk. The ability to assess a risk of a rare adverse event will depend on the availability of information concerning the background rates of the event in the population of interest. Such data on IS are limited in Central Asia, a region with a substantial diarrheal burden that could benefit from the introduction of rotavirus vaccination [25,26]. Our study is the first to document the incidence and epidemiology of childhood IS in the most populous country in Central Asia, using the internationally accepted case definition. Our estimates of IS incidence at 23 cases per 100,000 children is lower compared to rates reported from industrialized countries, such as the United States (50 per 100,000) [27], Australia (66 per 100,000) [28], and Europe (66-224 per 100,000) [29]. But our estimates are higher than figures from developing countries (18 cases per 100,000 in India and 0-17.8 per 100,000 in Bangladesh) [4], and similar to the incidence reported in middle-income Venezuela (24 per 100,000 children) [3]. However, comparisons of incidence rates for IS among different regions should be careful as these rates may be affected by variations in study methods, differences in genetic and lifestyle factors, and health care practices. Our estimates are based on the retrospective surveillance of cases, not allowing detection of all cases, thus resulting in the potential underestimation of incidence. Moreover, we calculated national rates using data from a single region, and if any differences in the incidence by region were to exist within the country, it would not have been reflected in our estimates.

Epidemiology of IS in Uzbekistan was similar to that described in other parts of the world. Previous reports specify that this condition is more frequent in males, with our study yielding a male to female ratio of 2:1. This ratio was reported to be varying widely across different regions, but all reports indicated predominance of males. For example, in geographically close Asia region, this ratio was reported to reach from 1.3:1 in Singapore [30] to 9:1 in India [31,32]. Most cases of IS occurred in infants between 3-6 months of age, which is the age when rotavirus vaccination is administered. This may challenge surveillance of intussusception following vaccination as a possible increase in cases of intussusception due to vaccination may coincide with its natural peak.. In Uzbekistan, no distinct seasonality of IS was detected, which is similar to reports from countries in the Americas [3,33] and Europe [34,35], In Uzbekistan, no distinct seasonality of IS was detected, which is similar to reports from countries in the Americas [3,33] and Europe [34,35]. Due to a small number of observations, we are unable to determine if a slight increase in the number of cases in April and May is truly seasonal.

Among symptoms of IS among hospitalized children, abdominal pain (97.0%) and the presence of an intestinal mass (97.0%) were most frequently reported. These symptoms develop during the first hours of IS. However, 87% of hospitalized cases were lethargic and 82% had blood detected during rectal examination, indicating a more progressed IS. The majority of children (87%) were hospitalized within 24 hours after symptom onset, and given good access to emergency health care services in Uzbekistan, most children would be hospitalized promptly. However, the time required to diagnose IS is highly dependent on clinicians' skills and the availability of the necessary equipment; so, a delayed diagnosis may lead to a more progressed clinical presentation. In our study, the most frequently used diagnostic method was plain abdominal radiography, followed by ultrasonography used in only a half of patients. Because all study hospitals had ultrasound machines, it is possible that the availability of personnel trained in transabdominal ultrasound techniques was limited, and physicians heavily relied on clinical indications for diagnosis. Only 50% of the cases in our study were managed successfully by air enema, whereas the remaining cases had a failed radiologic reduction and were admitted 24 hours after symptom onset. These patients needed surgical intervention, had a longer hospital stay, and a higher case-fatality rate. This is similar to reports from other countries that manage pediatric patients with a longer duration of symptoms [36]. Interestingly, no children in our study had repeated enema reductions, though it is possible that some patients may benefit from additional attempts. Among children who survived surgery, no post-operative complications or recurrences of IS were reported. However one died at surgery and two cases died immediately after surgery.

## Conclusions

In conclusion, our study provides useful information on the incidence and epidemiology of childhood IS in Uzbekistan. Because we did not conduct prospective surveillance in the current study, additional research using an active surveillance approach is needed to better determine incidence rates. Such surveillance with a standard case definition will not only provide better information for future post-marketing assessments on the safety of rotavirus vaccines, but will also raise awareness on IS among pediatricians and surgeons. More education on diagnosis and management of IS in pediatric population is needed in developing countries to help improve rates of successful outcomes.

## Competing interests

The authors declare that they have no competing interests.

## Authors' contributions

RL participated in the study design, contributed to the acquisition, analysis and interpretation of data, and drafted the manuscript. RK made substantial contributions to the acquisition and interpretation of data. EF conceived and designed the study, and helped to draft and revise the manuscript. All the authors read and approved the final manuscript.

## Pre-publication history

The pre-publication history for this paper can be accessed here:

http://www.biomedcentral.com/1471-2431/11/22/prepub
